# Tamoxifen metabolites treatment promotes ERα+ transition to triple negative phenotype *in vitro*, effects of LDL in chemoresistance

**DOI:** 10.1042/BSR20240444

**Published:** 2024-08-05

**Authors:** Andrea Muñoz-Ayala, Brenda Chimal-Vega, Nicolás Serafín-Higuera, Octavio Galindo-Hernández, Gladys Ramírez-Rosales, Iván Córdova-Guerrero, Luis Fernando Gómez-Lucas, Victor García-González

**Affiliations:** 1 Departamento de Bioquímica, Facultad de Medicina Mexicali, Universidad Autónoma de Baja California, Mexicali 21000, México; 2 Laboratorio Multidisciplinario de Estudios Metabólicos y Cáncer, Universidad Autónoma de Baja California, Mexicali 21000, México; 3 Facultad de Odontología Mexicali, Universidad Autónoma de Baja California, Mexicali 21000, México; 4 Departamento de Inmunología, Facultad de Medicina Mexicali, Universidad Autónoma de Baja California, Mexicali 21000, México; 5 Facultad de Ciencias Químicas e Ingeniería, Universidad Autónoma de Baja California, Tijuana 22390, México

**Keywords:** breast cancers, chemoresistance, metastasis, Nrf2, tamoxifen

## Abstract

Objective: Estrogen receptor-positive (ER+) breast cancer represents about 80% of cases, tamoxifen is the election neoadjuvant chemotherapy. However, a large percentage of patients develop chemoresistance, compromising recovery. Clinical evidence suggests that high plasmatic levels of low-density lipoproteins (LDL) could promote cancer progression. The present study analyzed the effect of LDL on the primary plasmatic active Tamoxifen’s metabolites resistance acquisition, 4-hydroxytamoxifen (4OH-Tam) and 4-hydroxy-N-desmethyl-tamoxifen (endoxifen), in breast cancer ERα + cells (MCF-7). Methods: Two resistant cellular variants, MCF-7^Var-H^ and MCF-7^Var-I^, were generated by a novel strategy and their phenotype features were evaluated. Phenotypic assessment was performed by MTT assays, cytometry, immunofluorescence microscopy, zymography and protein expression analysis. Results: MCF-7^Var-H^, generated only with tamoxifen metabolites, showed a critical down-regulation in hormone receptors, augmented migration capacity, metalloprotease 9 extracellular medium excretion, and a mesenchymal morphology in contrast with native MCF-7, suggesting the transition towards Triple-negative breast cancer (TNBC) phenotype. In contrast, MCF-7^Var-I^ which was generated in a high LDL media, showed only a slight upregulation in ER and other less noticeable metabolic adaptations. Results suggest a potential role of transcription factor nuclear factor erythroid 2-related factor 2 (Nrf2) in phenotypic differences observed among variants. Conclusion: LDL high or low concentrations during Tamoxifen´s metabolites chemoresistance acquisition leads to different cellular mechanisms related to chemoresistance. A novel adaptative cellular response associated with Nrf2 activity could be implicated.

## Introduction

Breast cancer (BC) is the most common malignancy in women worldwide. In 2020, BC had an approximate incidence of 2 million cases and generated an estimated 685,000 deaths (Global Cancer Observatory, 2022) associated with metastatic tumors in vital organs and tissues. Breast tumors can be classified by molecular subtypes, depending on the hormonal receptor expression [1], the main subtypes are Luminal A with Estrogen Receptor alpha (ERα)/Progesterone Receptor (PR) expression and no expression of human epidermal growth factor receptor type 2 (HER2); Luminal B with ERα/PR and HER2 expression; HER2 positive with HER2 overexpression and no expression of ERα/PR, and triple-negative (TNBC) with no hormonal receptors expression [2].

Approximately 80% of breast cancer tumors are ERα+, and for these cases, the neoadjuvant election treatment for premenopausal women is the selective estrogen receptor modulators (SERMS), mainly tamoxifen. Tamoxifen is a prodrug whose plasmatic primary active metabolites, 4-hydroxytamoxifen (4OH-Tam) and 4-hydroxy-N-desmethyl-tamoxifen (endoxifen), are generated by cytochrome P450 isoenzymes families such as CYP3A4 and CYP2D6 [3]. 4OH-Tam and Endoxifen have similar potency, possibly associated with the hydroxyl group at the C4 position in their structure [4]; although, endoxifen is the most abundant plasmatic metabolite in tamoxifen-treated patients [3]. Tamoxifen and its metabolites function as ERα antagonists in breast tissue, interfering with growth and proliferation pathways in cancer cells [4].

Tamoxifen treatment has been associated with a decrease in recurrence rate by 41% and a mortality rate of up to 31% [5]; however, nearly one-third of the patients relapse due to chemoresistance [6]. Several mechanisms responsible for tamoxifen resistance have been proposed in chemoresistant tumoral ERα+ cells; one of them corresponds to overexpression of sulfotransferase 1A1 (SULT1A), a phase II metabolism enzyme that inactivates 4OH-Tam and endoxifen by sulfation mechanism [7]. Other chemoresistance mechanisms are related to the ERα; for instance, loss or down-regulation of ERα expression by mutations, epigenetic modifications (ESR1), or post-translational alterations [8]. Changes in other hormonal receptors expression could occur as chemoresistant mechanism and/or associated with disease progression [9].

Changes in hormonal receptors expression among primary and metastatic tumors are frequently reported in clinical studies [10,11]. A meta-analysis that included 39 studies described a 19.3% change in ERα status, a 30.9% change in PR status, and a 10.3% change in HER2 status in metastatic tumors; the change from positive to negative status was the most frequent in this study [12]. Other meta-analysis evaluating hormonal status in BC brain metastasis in comparison with primary tumors reported a 42.6% discordance in any receptor status and individually a 17% in ERα status, 23% in PR status, and a 12% in HER2 status [13]. Although the causes of this phenomenon must be multifactorial, the effect of dyslipidemia in patients that undergo this complication has not been studied in detail.

High low-density lipoproteins (LDL) serum levels and low high density lipoprotein (HDL) serum levels or a high c-LDL/c-HDL ratio, commonly associated to an increased cardiovascular risk [14], has been also widely associated with an increased risk of developing BC cancer and BC cancer progression [15–19]. Focusing in LDL, it has been demonstrated *in vitro* that LDL cholesterol promotes proliferation and migration in ERα negative cell lines, while its effect on ERα+ cell lines remains debatable [19,20]. In animal models, it has been described a relationship between high LDL and total cholesterol levels and cancer progression [19]. *In vivo*, plasma LDL-C levels above 117 mg/dL have been positively associated with higher histological grade, higher proliferative rate and more advanced clinical stage [21]; on the other hand, tumors overexpressing LDLR, which allow them to capture more cholesterol, correlates to a more aggressive behavior and show a greater metastasis potential [22]. Although, the molecular mechanisms triggered by LDL high concentrations have not been described in detail in several contexts.

Metabolic adaptations are commonly related to cancer progression. It is common for tumoral cells to have increased free radical synthesis and, in consequence, augmented oxidative stress due to their accelerated metabolic activity [23]. Nuclear factor erythroid-2-related factor 2 (Nrf2), a leucine zipper transcription factor described as the redox homeostasis master regulator, plays an essential role in managing oxidative stress in tumoral cells [23,24] in addition to increasing metastatic capacity [25] and potentially chemoresistance. In turn, this phenotype could be triggered by high LDL concentration.

Therefore, this study aimed to evaluate the effect of LDL on tamoxifen resistance generation in a cellular ERα+ breast cancer model. For the first time, the induced chemoresistance was reached through treatment with primary plasmatic tamoxifen-derived metabolites (4-OH Tam and endoxifen), promoting the transition to a TNBC phenotype-like. However, contrary to what was expected, this phenotype was not observed under co-incubation of metabolites with a high LDL concentration (75 µg/ml). Elucidating LDL role in ERα BC tamoxifen chemoresistance acquisition and the molecular mechanisms involved is critical to stablish the most suitable disease management and to propose new therapeutic targets.

## Materials and methods

### Materials

Cell culture reagents were purchased from Thermo-Fisher (Carlsbad, CA, U.S.A.), tissue culture plates and other plastic materials were obtained from Corning Inc. Tamoxifen metabolites 4OH-Tam and endoxifen were obtained from Santa Cruz Biotechnology with a purity greater than 98%. Tunicamycin (Tum) was obtained from Sigma-Aldrich. Buffers and Dimethylthiazol-2-yl-2,5-diphenyltetrazolium bromide (MTT) were obtained from Merck (Darmstadt, Germany). Monoclonal antibodies anti-Fas (1:250), anti-LDH-A (1:400), anti-PCNA (1:2500), anti-Nrf2 (1:300), anti-SNAI-1 (1:250) anti-β-actin (1:300), anti-FAK (1:300), Anti-p-FAK (1:400), anti-Ki67 (1:900), anti-HER2 (Neu) (1:200), anti-ER alpha (1:250), anti-PR (1:400), anti-HO-1 (1:500), anti-SULT1A1 (1:400), and anti-NQO1 (1:500) were obtained from Santa Cruz Biotechnology. LDLR (1:800) from Abcam. Anti-mouse secondary antibodies coupled to Horseradish peroxidase from Thermo-Fisher. Chemiluminescence detection was performed using Immobilon Western kit (Millipore, MA, U.S.A.) and X-ray film for some blots, for others Bio-Rad ChemiDoc XRS+ was used and a digital image was obtained.

### Cell culture

MCF-7 cells obtained from American Type Culture Collection (ATCC), were grown in Dulbecco’s modified Eagle’s medium (DMEM) supplemented with 10% fetal bovine serum (FBS), 50 U/ml penicillin and 50 µg/ml streptomycin and insulin (0.57 UI/ml). Cells were maintained at 37°C with 90% humidity and 5% CO_2_.

### Chemoresistance generation

Through the development of this protocol, two chemoresistant variants were generated. MCF-7^Var-H^: the treatment was performed with equimolar 1 µM concentration of tamoxifen metabolites 4OH-Tam and endoxifen in culture media for 30 days. Later, the concentration was increased to 2 µM for both metabolites in culture media for 15 days. Cells were maintained under 50 nM concentration of both metabolites during cellular expansion and recovery period. MCF-7^Var-I^: treatment was followed as referred to above for MCF-7^Var-H^ plus 75 µg/ml of LDL for 45 days. Cells were maintained in 50 nM concentration of both metabolites and 7.5 µg/ml of LDL for expansion. Chemoresistance acquisition in both variants was confirmed through MTT assay. In each experimental panel, parental MCF-7 cells were used as a control.

### LDL isolation and fluorescent labeling

Human plasma samples were obtained from a healthy donor who signed an informed consent. The protocol was designed and carried out according to the Declaration of Helsinki and registered in the Research Ethics Committee of Facultad de Medicina Mexicali (FMM/CEI-FMM/006/2022-1). Briefly, plasma density was initially adjusted to density 1.019 g/ml by adding KBr and then centrifuged at 345,000 ***g*** for 160 min at 4°C. Later, fraction <1.019 g/ml was discarded, and the remaining plasma was adjusted to 1.053 g/mL density. It was centrifuged at 377,000 ***g*** for 200 min. Finally, <1.053 g/ml fraction was recovered, dialyzed against 150 mM NaCl/EDTA 0.024 mM, and filtered through 0.45 μm. In the LDL fraction, protein concentration was measured with the bicinchoninic acid assay (BCA), and the determination of LDL-cholesterol was carried out (Spinreact). A defined volume of buffer (NaCl 150 mM, EDTA 0.024 mM) corresponding to a specific dose was evaluated as a control for the experimentation. The quality of the isolates was evaluated by characterization of apolipoproteins apoB-100 and apoA1 (Supplementary Figure S1).

Labeling of LDL fraction was carried out with the dilC18 probe (D3911), which is incorporated into the phospholipid monolayer, through incubation of 10 µl of the probe (2 mg/ml) for each 1 mg of protein-LDL for 18 h at 37 °C, obtaining dil-LDL. The solution density was adjusted to a 1.053 g/ml and centrifuged at 377,000 ***g*** for 200 min to recover fluorescent LDL. The fraction was recovered and dialyzed against PBS. The experiments evaluated a range of 0–75 µg/ml.

### dil-LDL cytometer assays

Before internalization experiments, MCF-7 and cell variant cultures at 90% confluence were incubated in a FBS-free culture medium. Two hours fasting later, cells were treated under diferent dil-LDL concentrations (0–75 µg/ml) for 24 h. Later, cells were washed twice with PBS and recovered in a 200 µl volume. Cellular characterization was performed in a Beckman-Coulter cytometer Cytoflex. Employing the PC7-A channel to record the dil-LDL fluorescence based on a previous work, 30,000 events were registered [26].

### Cell viability assay

Cell viability was assessed using an MTT reduction assay. Cells were seeded into 96-well plates with a density of 20,000 cells/well and allowed to grow to 75% confluence. The DMEM-supplemented medium was replaced with Opti-MEM and incubated at 37°C for 2 h; later, several treatments were added (0.125–16 µM of 4-OH Tam or Endoxifen) and incubated for 72 h. The next step was the addition of 30 µl MTT (2.1 g/ml) in Opti-MEM and incubation at 37°C for 4 h. Formazan crystals generated by mitochondrial activity were dissolved with a lysis buffer (20% SDS, 50% N, N-dimethyl-formamide, pH 3.7). A lecture on absorbances at 590 nm was performed 12 h later. The IC_50_ of each metabolite was calculated with GraphPad Prism 8.

### Wound-healing assay

Cells were placed in a 6-well plate under a density of 400, 000 cells/well and allowed to grow to 90–95% confluence, forming a cellular monolayer. The DMEM-supplemented medium was replaced with the DMEM experimental medium, and cells were incubated at 37°C for 2 h. Later, cells were treated with mitomycin C for 2 h to prevent cell proliferation. The monolayer was scratched with a pipette tip, washed with PBS to remove floating cells, and treatments were added. Cells were allowed to migrate for 72 h at 37°C. Each well was fixed with paraformaldehyde, stained with Blue Coomassie, and photographed using a Motic Images Plus 3.0 camera coupled to an optical inverted microscope. Results were processed using ImageJ software, and statistical analysis was calculated using GraphPad Prism 8.

### Cellular lysates and Western blot analysis

Cells were seeded into 6-well plates with a density of 400,000 cells/well and allowed to reach 90–95% confluence. Next, cells were incubated under the specific treatments. Cells were washed with PBS and lysed with a protein lysis buffer supplemented with protease and phosphatase inhibitors. Sample proteins were quantified by the BCA assay. Samples (25 µg/lane) were analyzed by SDS-PAGE on 8–12% gels and further transferred to PVDF membranes. Later, membranes were blocked in TBS/0.1% tween (TBST)/5% low-fat milk at 37°C for 1 h and incubated at 4°C overnight with the corresponding primary antibody. Next, membranes were washed with TBST and incubated for 120 min at 37°C with the corresponding horseradish peroxidase-conjugated (HRP) secondary antibodies. Membranes were washed with TBST, and the HRP activity was detected with the Immobilon Western Kit (Millipore, MA, U.S.A.). Analysis of immunoblot films was made with the ImageJ software (NIH, Maryland, DC, U.S.A.).

### Optical microscopy

Cells were photographed using a Motic Images Plus 3.0 camera coupled to an optical inverted microscope.

### Zymography analysis

Variants and MCF-7 cells were seeded at a density of 200,000 cells/ml. Cellular cultures were incubated under 90% confluency under the specific schemes, and the extracellular medium was collected. Volumes of 40 µl non-heated conditioned medium samples were mixed with 5× sample buffer (0.313 M Tris pH 6.8, 10% SDS, 50% glycerol, and 0.05% bromophenol blue) and loaded on 8% polyacrylamide gels copolymerized with gelatin (1% *w*/*v*). Gels were rinsed twice with 2.5% Triton X-100 and then incubated in development buffer (50 mM Tris-HCl pH 7.4, 10 mM CaCl_2_, and 0.02% NaN_3_) for 40 h at 37 °C. Gels were fixed and stained with 0.25% Coomassie Brilliant Blue G-250 in 10% acetic acid and 30% methanol. Proteolytic activity was detected as clear bands against the background stain. In these experiments, β-actin as a reference for the amount of protein was used as a loading control.

### Nrf2 localization

We performed the analysis of Nrf2 cellular localization. Briefly, cells were seeded on slides placed in untreated 6-well dishes (400,000 cells/well) and allowed to reach 60–70% confluence. Cells were incubated under several treatments for 24 h at 37°C. Later, cells were washed with filtered PBS and fixed with 2% paraformaldehyde/PBS. Next, cells were treated with a 0.01% triton solution for 5 min and blocked with 2% bovine albumin/PBS for 30 min. Cells were incubated with anti-Nrf2 antibody overnight at 4°C. Later, cells were washed with PBS and incubated with a FITC-coupled secondary antibody. Subsequently, counterstaining was carried out with Propidium Iodide. Finally, the cells were observed using the green and red filter in an EPI-fluorescence microscope (Axio VertA.1, Zeiss, Göttingen, Germany).

### GEPIA analysis

We used the GEPIA database (http://gepia.cancer-pku.cn/about.html) to evaluate the Nrf2 role in breast cancer. We analyzed the mRNA expression levels of NFE2L2 (Nrf2 coding gene, Gene ID: 4780) and their relationship with overall survival in BC patients using GEPIA which collects information from the TCGA (The Cancer Genome Atlas) [27] and GTEx (Genotype-Tissue-Expression) [28].

### Molecular docking

For molecular docking experimentation, the atomic coordinates of protein SULT1A1 (PDB ID: 4GRA) with a resolution of 2.56 Å were evaluated. The structures of tamoxifen (CID:10540-29-1), 4OH-Tam (CID: 112093-28-4) and endoxifen (CID:11002528-0) were obtained from the PubChem Database. The protein structure was prepared, waters and small molecules were removed. The ligands and protein were 3D-protonated and energy minimization was carried out by employing Molecular Operating Environment software (MOE) using default parameters (Placement: Triangle Matcher, Rescoring 1: London G, AMBER99 forcefield). Each ligand was generated up to different conformations and protein was visualized with ligand interactions implemented in MOE. Estradiol was used as a natural ligand (CHEBI: 23965), obtained from ChEBI Database.

### Statistical analysis

Data are expressed as mean± SD. The IC_50_ and ANOVA probes were calculated in GraphPad Prism 8.

## Results

### ERα+ cells internalize LDL

**Figure 1:**
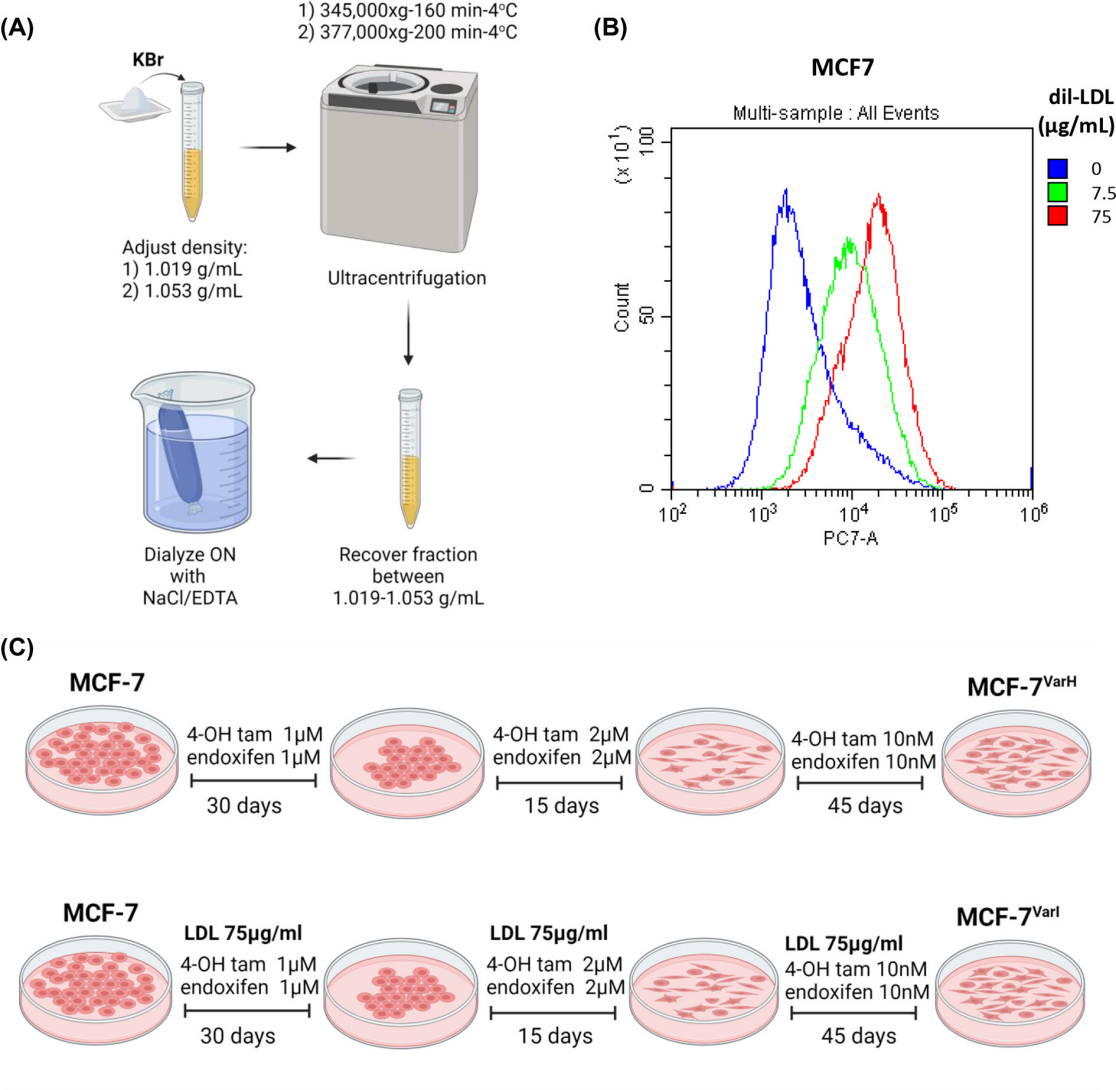
LDL internalization and chemoresistance induction in MCF-7 cells (**A**) Scheme corresponding to LDL isolation by KBr method. (**B**) Evaluation of dil-LDL internalization on parental MCF-7 cells (0, 7.5, 75 µg/ml) through flow-cytometry. (**C**) Scheme of chemoresistance protocol for generation of MCF-7^Var-H^ and MCF-7^Var-I^ cell variants. (A) and (C) were created in BioRender.com.

We focused on the LDL endocytosis capability of the ERα+ cell model (MCF-7 cells). LDL isolation was performed by the KBr ultracentrifugation method (Figure 1A) and validated through the evaluation of protein targets apoA-1 and apoB-100 on LDL enriched fraction, but also in VLDL, HDL enriched fractions and in plasma-free fraction. Results verified the appropriate enrichment of LDL in the desired fraction (Supplementary Figure S1). The yields in the LDL fraction were characterized by protein determination (0.76 ± 0.13 µg/µl) and LDL-cholesterol quantification (1.190 ± 0.39 mg/dL). LDL was labeled with a fluorescent dil-C18 probe, obtaining dil-LDL particles based on previous reports [29,30] (Section 2.4). Then, MCF-7 cells were treated for 24 h with dil-LDL (0–75 µg/ml) and evaluated by flow cytometry, with results suggesting MCF-7 cell internalization (Figure 1B).

For chemoresistance induction, for the first time, MCF-7 cultures were incubated under an equimolar ratio of 4OH-Tam and endoxifen, the primary plasmatic tamoxifen-derived metabolites. In this regard, we generated two cellular variants (Figure 1C); for the variant denominated MCF-7^Var-H^, we treated MCF7 cells with 1 µM of 4OH-Tam and 1 µM Endoxifen for a 30-day period; the metabolites concentration was increased to 2 µM and treatment was continued for 15 days. Later, cell cultures remained in a proliferation and recovery period for 45 days with 50 nM of metabolites (Figure 1C). For MCF-7^Var-I^, cells were treated with 1 µM of 4-OH Tam and 1 µM Endoxifen for a 30-day period and concomitant 75 µg/ml concentration of LDL. Metabolite concentration was increased to 2 µM, and treatment was continued for 15 days; LDL concentration remained unchanged. After treatment was finished, cells remained in a proliferation and recovery period for 45 days with 50 nM of metabolites plus 7.5 µg/ml of LDL (Figure 1C).

### Chemoresistance induction by tamoxifen’s metabolites

**Figure 2:**
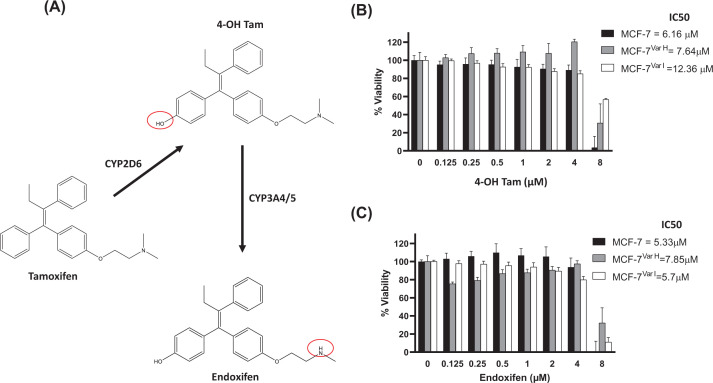
Chemoresistance evaluation in MCF-7 derived cellular variants (**A**) Tamoxifen metabolites chemical structures and *in vivo* synthesis representation by CYP isoenzymes, structures were generated with ChemDraw. Cell viability for 4OH-Tam (**B**) and Endoxifen (**C**) evaluated by MTT assay in a metabolite range of 0.25–8 µM. Treatments were performed during 72 h. Inserts showed values of inhibitory concentration 50 (IC_50_) in cellular variants.

Later, we determined the chemoresistance through half-inhibitory concentration (IC_50_) for 4OH-Tam and endoxifen on cellular variants regarding parental cells, using the MTT assay. A range of 0.125–8 μM metabolite concentration was employed and treatments were maintained for 72 h (Figure 2B,C). The IC_50_ values of 4OH-Tam (7.64 µM) and endoxifen (7.85 µM) were higher for MCF-7^Var-H^ than for the parental cells, whose values were 6.16 and 5.33 µM, respectively (Figure 2B,C).

On the other hand, MCF-7^Var-I^ showed IC_50_ values of 12.36 and 5.7 µM for 4OH-Tam and endoxifen, respectively (Figure 2B,C), a different behavior from the observed one in MCF-7^Var-H^. For the first time, we reported the chemoresistance acquisition under equimolar concentrations of the main pharmacological active tamoxifen-derived metabolites and the influence of high LDL concentrations (75 µg/ml).

### Important hormonal receptor down-regulation associated with chemoresistance in MCF7^VarH^ cells

**Figure 3:**
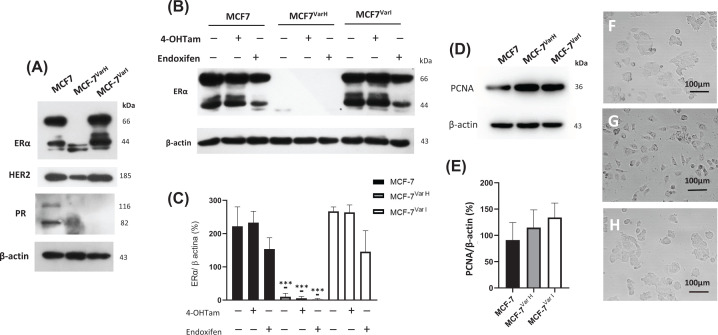
Chemoresistance is associated with a transition of ER+ to TNBC like phenotype (**A**) Evaluation of hormonal receptor expression, ERα, PR and HER2 by western blot. β-actin was used as a loading control in parental, MCF-7^Var-H^, and MCF-7^Var-I^ cells. (**B**) ERα expression under 4OH-Tam and endoxifen treatment (8 µM) in three cellular variants. (**C**) Densitometry analysis of ERα; results are reported as mean ± SD (*n* = 3); ***P<0.001 regarding respective control. (**D**) Expression of PCNA in variants MCF-7, MCF-7^Var-H^, and MCF-7^Var-I^ cells. (**E**) Semiquantitative analysis of PCNA in three cellular variants. Optical microscopy images corresponding to MCF-7 (**F**), MCF-7^Var-H^ (**G**), and MCF-7^Var-I^ cells (**H**), images were taken at low cellular density with 40× objective.

Several clinical studies have described the association between chemoresistance acquisition and the down-regulation of hormonal receptors in breast tumors [11,31]. Then, hormonal receptor status in chemoresistant cellular variants was evaluated. We analyzed the expression of ERα, HER2, and progesterone receptor (PR). A significant down-regulation in the three hormonal receptor expression levels in the MCF7^VarH^ was registered (Figure 3A). However, MCF7^VarI^, obtained by co-incubation of MCF-7 cells with tamoxifen’s metabolites and LDL, did not display these drastic changes (Figure 3A). Considering that the 4-OH Tam and endoxifen major pharmacological effects are related to ERα inhibition, we decided to evaluate the receptor levels in cellular variants under treatment with tamoxifen metabolites (8 µM), a standard concentration according to IC_50_ values. Interestingly, down-regulation of ERα remains in MCF7^VarH^ cells even under 4OH-Tam and endoxifen treatment, and its effect on MCF-7 and MCF7^VarI^ appears to be not statistically significant (Figure 3B,C).

As a complementary characterization, proliferation cell markers were evaluated. Expression levels of PCNA related to cell division were higher in MCF-7^Var-H^ and MCF-7^Var-I^ cells with regard to MCF-7 parental (Figure 3D,E). Likewise, we observed an important morphological change in MCF-7^Var-H^, these cells acquired a mesenchymal-like morphology distinct to epithelial morphology of MCF-7 and MCF-7^Var-I^ cells (Figure 3F–H). The down-regulation of hormonal receptors, PCNA levels, and the mesenchymal morphology led us to suggest the induction of TNBC-like phenotype in MCF-7^Var-H^ variant.

### MCF-7^Var-H^ cells showed enhanced migratory capability *in vitro* and augmented metalloproteinase secretion

**Figure 4:**
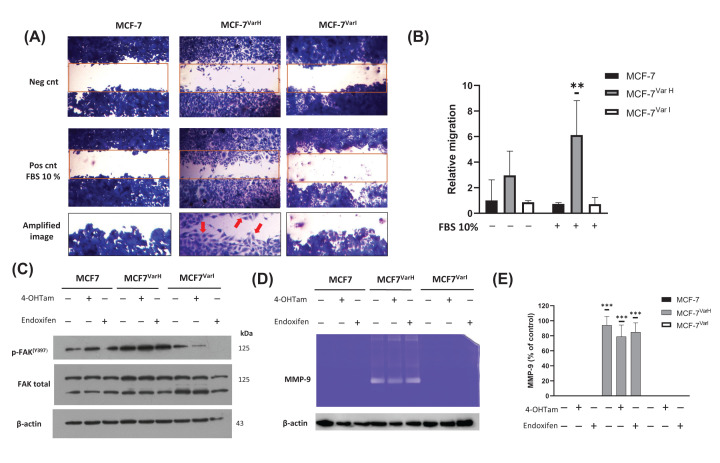
Characterization of cell migration and invasion capability in chemoresistant cellular variants (**A**) Representative images of the wound-healing assay in MCF-7, MCF-7^Var-H^, and MCF-7^Var-I^ cells under FBS stimuli (10%) and respective controls. In an amplified image of cells, lamellipodia and filopodia were observed in MCF-7^VarH^ migrating cells, indicated by a red arrow. (**B**) Wound-healing results expressed as percentage respect to controls in parental, MCF-7^Var-H^, and MCF-7^Var-I^ cells. Mean values are presented (*n*=3, mean ± SD); **P<0.005 respect to MCF-7 control. (**C**) Under tamoxifen-derived metabolites treatment (8 µM), evaluation of FAK activation (p-FAKY^397^) by Western blot. β-Actin was used as a loading control. (**D**) Representative image of MMP-9 activity in extracellular media of cellular variants, under 4-OHTam and endoxifen treatment (8 µM). (**E**) Statistic evaluation of MMP-9 activity in extracellular media of three biological replicates, ***P<0.0001 with respect to controls.

TNBC tumors are associated with aggressive behavior and a poorer prognosis than other molecular subtypes. Then, we decided to evaluate the metastatic potential in resistant cellular variants, mainly in MCF-7^Var-H^, to determine the potential acquisition of TNBC-like features. First, we assessed migration capability through wound healing assay (Figure 4A), and we found that MCF-7^Var-H^ showed high migratory capability to the wound area under FBS 10% stimuli. Meanwhile, neither MCF-7 nor MCF-7^Var-I^ showed migratory capacity under the evaluated conditions (Figure 4A,B). We identified in wound healing assay a mesenchymal morphology in MCF-7^Var-H^ migrated cells, mainly on the migration fronts (Figure 4A), probably associated with rearrangements in their cytoskeleton.

In aggressive tumors, the Focal Adhesion Kinase (FAK) pathway is often hyperactivated, promoting stromal remodeling and inducing tissue stiffness, cell proliferation, survival, and chemoresistance [32]. Then, we evaluated FAK expression and activation (p-FAKY^397^); data suggest FAK activation (p-FAKY^397^) in MCF-7^Var-H^ (Figure 4C), wherein the data corroborate a potential greater malignancy in MCF-7^Var-H^ compared to MCF-7^Var-I^ and parental cells. Even more, our previous evidence showed that FAK pathway activation was associated with chemoresistance in TNBC cells [26]

In a complementary way, we evaluated the extracellular matrix degradation capability of the chemoresistant variants and parental cells, a phenomenon associated with metastasis processes [33]. In the first focus, matrix metallopeptidase 9 (MMP-9) activity was evaluated in the extracellular media of cellular variants. In an important way, we observed a high activity of MMP-9-mediated collagen degradation only in MCF-7^Var-H^ cells (Figure 4D,E); this phenomenon was not affected by 4OH-Tam or endoxifen treatments (8 µM) (Figure 4D), a concentration close to the IC_50_ for both metabolites. This phenomenon suggests a high invasive capability in the MCF-7^Var-H^ despite pharmacological treatment. Even more, it has been described that metabolic adaptations can occur, for instance, in targets involved in lipoprotein metabolism, associated with an increase in tumor aggressiveness [34].

### Metabolic evaluation and the LDL role in chemoresistance acquisition

**Figure 5:**
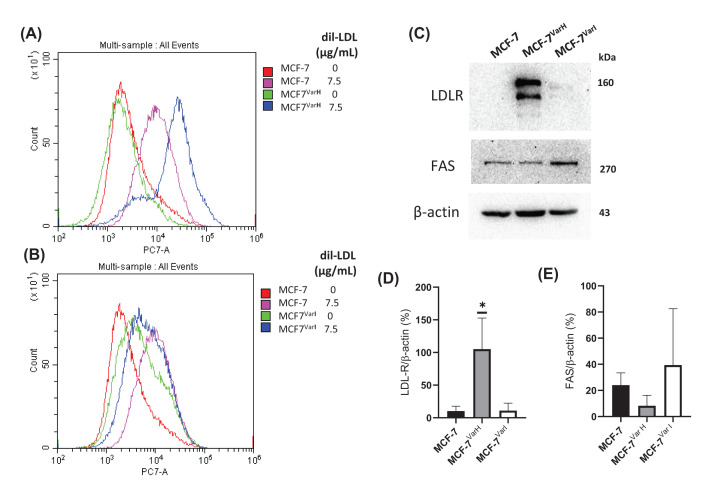
Characterization of lipid metabolism in chemoresistant variants LDL internalization in MCF-7^Var-H^ (**A**) and MCF-7^Var-I^ (**B**) regarding MCF-7 parental cells. Cell cultures were dil-LDL (7.5 µg/l) treated for 24 h. **C**) Expression of the protein targets LDLR and FAS in MCF-7, MCF-7^Var-H^ and MCF-7^Var-I^ cells. Densitometry analysis of LDLR (**D**) and FAS (**E**), mean values are presented (*n*=3, mean ± SD) and expressed as % of control, *P<0.01. β-Actin was used as a loading control.

We evaluated LDL internalization in cellular variants after 7.5 µg/ml dil-LDL treatment (24 h). Results suggest a slightly higher capability for LDL endocytosis in MCF-7^Var-H^ (97.12%) cells in comparison with MCF-7 (80.46%) (Figure 5A). Indeed, the lowest levels of LDL internalization were recorded in variant MCF-7^Var-I^ (7.82%) (Figure 5B). In an attempt to explain this behavior, we determined the LDLR expression. We observed a correspondence of LDL internalization capability and LDLR expression in chemoresistant variants (Figure 5C,D).

A down-regulation trend in Fatty Acid Synthase (FAS) was registered in MCF-7^Var-H^ as well as an increase in the MCF-7^Var-I^, suggesting a potential different rearrangement in lipid metabolism in both variants (Figure 5C,E). Other metabolic changes related to chemoresistance could take place; it is common for chemoresistant tumoral cells to have an enhanced antioxidant and detoxification capacity [23]; this response may be related to the activation of specific transcriptional factors.

### Overexpression of transcription factor Nrf2 could be associated with phenotypic changes

**Figure 6:**
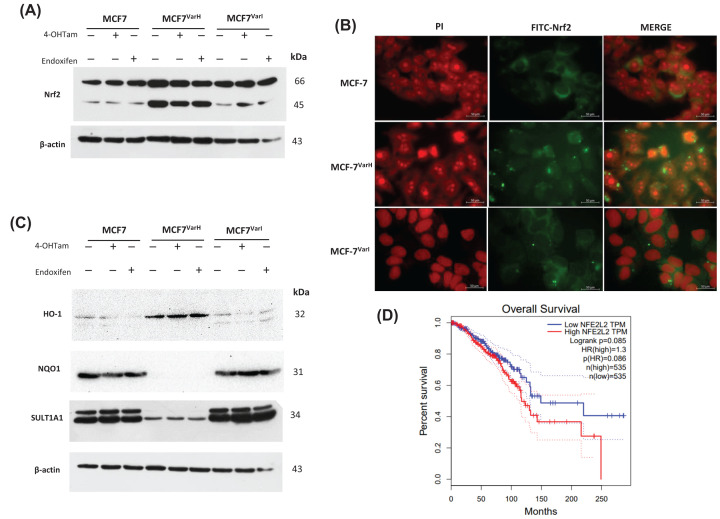
Overexpression of Nrf2 could be associated with chemoresistance induced through treatment with tamoxifen derived metabolites in MCF-7^Var-H^ (**A**) Western blot of Nrf2 expression in variants MCF-7, MCF-7^Var-H^, and MCF-7^Var-I^ under tamoxifen derived metabolites treatment (8 μM). β-Actin was used as a loading control. (**B**) Localization of Nrf2 in MCF-7, MCF-7^Var-H^, and MCF-7^Var-I^ cells. Images showed nuclei stained with propidium iodide (PI) (red), FITC-Nrf2 (green) and the merge. Scale bar corresponds to 50 µm. (**C**) Characterization of the expression of HO-1, NQO1 and SULT1A1 in MCF-7, MCF-7^Var-H^, and MCF-7^Var-I^ cells under the tamoxifen metabolites treatment (8 µM). β-Actin was used as a loading control. (**D**) Overall survival plot for low and high expression of NFE2L2 (Nrf2 coding gene) in BC patients from GEPIA.

Nrf2, a transcription factor involved in the management of cellular redox conditions [35], has been associated with chemoresistance and metastasis [36,37], and could be related to changes in hormone receptor status (personal communication). Critically, we found a Nrf2 overexpression in MCF-7^Var-H^ cells (Figure 6A) and this behavior was not influenced by tamoxifen-derived metabolites treatment (Figure 6A). Likewise, our data suggest an overexpression of a lower molecular weight (45 kDa) Nrf2 isoform in MCF-7^Var-H^, and this phenomenon is also not influenced by metabolites treatment (Figure 6A). Lower molecular weight Nrf2 bands have been previously reported *in vitro* [38–40] and *in vivo* in pulmonary tumors [39]; however, in breast cancer cells have not been reported.

In a complementary way, we characterized the Nrf2 cellular localization; we employed the monoclonal Nrf2 antibody and FITC coupled secondary antibody, and nuclear colocalization was determined by the use of propidium iodide. Results suggest nuclear and cytoplasmic Nrf2 localization in MCF-7^Var-H^ cells, compared with MCF-7^Var-I^ and parental cells (Figure 6B) wherein its localization is mostly in cytoplasm. Several controls were used in these experiments (Section 2.11). Therefore, results correspond with data of protein expression (Figure 6A).

In order to broaden the impact of Nrf2 on chemoresistance development, we characterized protein targets dependent on Nrf2 activity. In this case, we found a differential expression of Nrf2 most representative targets such as heme oxygenase-1 (HO-1) and NAD(P)H dehydrogenase [quinone] 1 (NQO1) (Figure 6C). In MCF-7^Var-H^ cells, HO-1 was overexpressed while NQO1 was down-regulated compared with MCF-7 cells. For its part, in MCF-7^Var-I^ HO-1 and NQO1 expression levels remain similar to those in MCF-7, despite tamoxifen-derived metabolites treatment (8 µM). This phenomenon suggests the presence of a specific antioxidant profile adapted to cell survival in chemoresistant MCF-7^Var-H^ cells (Figure 6C).

Subsequently, we evaluated the expression of SULT1A1, an enzyme whose expression is influenced by Nrf2 activity and has been related to 4OH-Tam and endoxifen inactivation by a sulfate group addition reaction [41]. Contrary to what was expected, a SULT1A1 down-regulation was observed in MCF-7^Var-H^ cells (Figure 6C; Supplementary Figure S3), this response is not influenced by metabolites treatment (8 µM) in any of the variants. In complementary way, we performed a molecular docking characterization to evaluate SULT1A1 affinity for tamoxifen metabolites in comparison with estradiol, one of its known target molecules, founding a lower affinity (Supplementary Figure S4). Data suggest Nrf2 regulates the expression of necessary genes in the MCF-7^Var-H^ cells based on a specific gene regulation condition, suggesting a specific adaptive cellular response.

Considering the potential implications of Nrf2 overexpression, we assessed the role of Nrf2 in breast cancer patient’s prognosis databases. Therefore, we evaluated the role of Nrf2 expression levels in breast cancer patients using the GEPIA database (http://gepia.cancer-pku.cn/about.html). The data showed a marked relationship between high expression of Nrf2 and a lower overall survival (Figure 6D).

The activation of the Nrf2 pathway may be related to chemoresistance development and augmented metastasis-related processes in the MCF-7^Var-H^ cells, even more so with the transition toward a TNBC phenotype. In this regard, we registered an increment in Nrf2 expression dependent on the concentration of Tunicamycin (Tuni) (0–4 µg/ml) in the cellular variants (Supplementary Figure S5A,C), a compound that inhibits protein N-glycosylation in the endoplasmic reticulum (ER) [42], triggering the unfolded protein response (UPR) and activating Nrf2 [43]. Tuni concentrations as low as 0.25 μg/ml could upregulate Nrf2. Subsequently, in the evaluation of hormonal receptors ERα and HER2 expression in the cellular variants, we observed an inverse relationship among Tuni dose and hormonal receptor expression (Supplementary Figure S5B,D).

## Discussion

High LDL levels have been associated with BC development and progression in many *in vitro* [18], *in vivo* [19], and clinical studies [44]; however, the potential LDL impact on chemoresistance in ER+ BC cells has not been described clearly. We developed two chemoresistant cellular variants by co-incubation of ER+ MCF7 cells with endoxifen and 4-OH tam. Although several protocols have been described for tamoxifen resistance generation in MCF-7 cells [45] it is the first time that chemoresistance is achieved under an equimolar treatment scheme of the two primary plasmatic tamoxifen’s active metabolites. MCF-7^Var-I^ was obtained by maintaining high LDL concentrations (75 µg/ml) in culture media during chemoresistance acquisition period, and MCF-7^Var-H^ was obtained with metabolites treatment only (Figure 1C, Section 2.3). This strategy was designed to evaluate the LDL effect on chemoresistance development, simulating the mechanisms triggered in ER+ breast cancer patients with high LDL plasma levels during tamoxifen treatment.

We determined chemoresistance acquisition by MTT cell viability assays in MCF-7^Var-H^ and MCF-7^Var-I^ by comparing their IC_50_ metabolites values with those of MCF-7 parental cells (Figure 2B,C). Among differences observed in cellular variants, one of the most noticeable was that MCF-7^Var-H^ showed a significant knockdown in the hormonal receptors ERα and HER2. In contrast, although not significant, MCF-7^Var-I^ showed a slight increase in the expression of ERα and HER2 compared to MCF-7 cells (Figure 3A–C).

MCF-7^Var-H^ apparently showed diminished estradiol (E2)-ERα mediated signaling. Furthermore, another target of E2 is the G protein-coupled receptor 30 (GPR30) [46]; our data suggest a knockdown in GPR30 expression on MCF-7^Var-H^ (personal communication), providing evidence that in these chemoresistance cells the response mediated by E2 is down-regulated.

**Figure 7:**
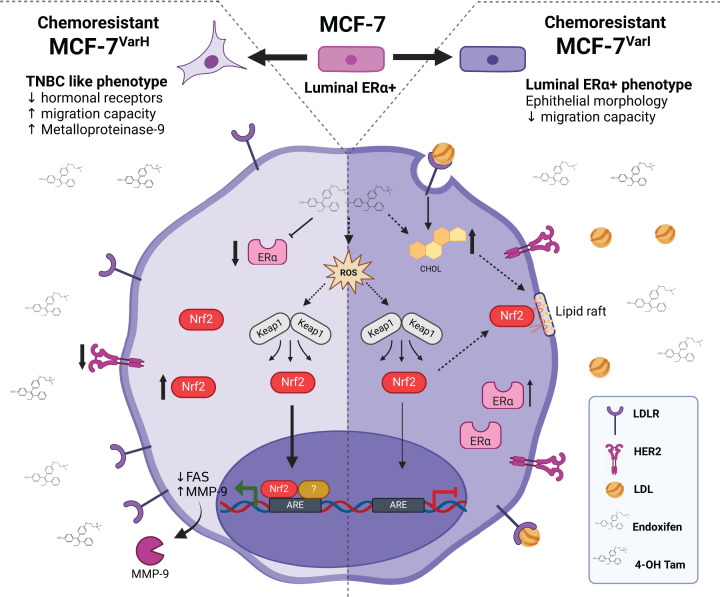
Phenotypic differences between MCF-7^Var-H^ and MCF-7^Var-I^ MCF-7^Var-H^ (left side) generated by treating the MCF-7 cell line with equimolar concentrations of 4-OH Tam and Endoxifen showed decreased hormonal receptors, increased migration capacity, and increased MMP-9 secretion, features similar to those observed in TNBC. Our proposed mechanism involves an increased Nrf2 expression and activity favored by metabolites ROS generation. We suggest that Nrf2 activity promotes proteins related to migration and invasion processes transcription. MCF-7^Var-I^ (right side) generated by treating the MCF-7 cell line with equimolar concentrations of 4-OH Tam and Endoxifen in addition to 75 mg/dl of LDL cholesterol maintained its luminal ER+ phenotype related to an epithelial morphology and a low migration capability. We proposed that high intracellular concentrations of cholesterol, generated by LDL treatment and the effect of tamoxifen metabolites in LDL intracellular traffic, favor Nrf2 interaction with cav-1 in lipid rafts, preventing its nuclear accumulation and transcriptional activity. Dashed lines refer to proposed mechanisms; solid lines refer to observed phenomena in variants. Triple negative breast cancer (TNBC), low-density lipoprotein receptor (LDL), human epidermal growth factor receptor type 2 (HER2), estrogen receptor α (ERα), cholesterol (CHOL), fatty acid synthase (FAS), metalloproteinase-9 (MMP-9), reactive oxygen species (ROS), antioxidant response element (ARE). Created with BioRender.com

Moreover, MCF-7^Var-H^ showed enhanced proliferation corroborated by PCNA (Figure 3D,E) and Ki67 (Supplementary Figure S2) by Western blot compared with MCF-7^Var-I^ and MCF-7. Another visible difference was the morphology, MCF-7^Var-H^ acquired a mesenchymal-like phenotype, while MCF-7^Var-I^ conserved an epithelial one (Figure 3F–H). Those results led us to propose the similarity of MCF-7^Var-H^ variant with TNBC cells, which are commonly related to a basal phenotype and have a greater metastatic capability compared with ER+ BC cells [47]. To corroborate this proposal, we evaluated some processes associated with metastasis i*n vitro*, such as migration capability and MMP-9 activity in extracellular media. As expected, MCF-7^Var-H^ had an increased migratory capability compared with MCF-7^Var-I^ and MCF-7 (Figure 4A,B). Indeed, the mesenchymal morphology of MCF-7^Var-H^ cells at the wound edge was evident. In addition, only MCF-7^Var-H^ metalloproteinase secretion was detected in zymography, which was not affected by treatment with Tamoxifen metabolites (Figure 4D,E). Therefore, MCF-7^Var-H^ acquired a TNBC like phenotype, while MCF-7^VarI^ maintained its luminal cell behavior and characteristics (Figure 7).

In another instance, we registered significant changes in lipid metabolism targets in both variants. MCF-7^VarI^ showed an augmented expression of FAS which catalyzes palmitic acid syntheses using acetyl-CoA, malonyl-CoA and NADPH as cofactor [48], suggesting active lipogenic metabolism in this variant. MCF-7^VarI^ also exhibited a significant low dil-LDL internalization of only 7.82%, that could be related to a negative feedback mechanism due to exposure to high LDL concentrations during chemoresistance acquisition. For its part, MCF-7^VarH^ expressed statistically significant higher LDLR expression levels than MCF-7 and showed a corresponding enhanced LDL internalization after treatment with 7.5µg/ml dil-LDL (Figure 5). Previously, increased LDL internalization and neutral lipid storage were described in the TNBC cell line MDA-MB-231 compared with MCF-7 [49]. This evidence suggests again an ER+-TNBC transition in MCF-7^VarH^ cells.

Furthermore, it has been described that SERMS such as tamoxifen and its metabolites can alter lipidic metabolism in cells independently of their action on ERα [50]. SERMS can favor the activation of SREBP2 and, in turn, inhibit the intracellular traffic of LDL-cholesterol, increasing cellular cholesterol accumulation [51]. Cholesterol accumulation facilitates the generation of oxysterols such as 27-hydroxy-cholesterol [34], metabolite related to ERα cell proliferation in low E2 environments [34,52]. E2-deprived MCF-7 cells exert an increased response to oxysterols in contrast with MCF-7 native cells, which favors REα mediated transcription [53]. To explain the phenotypic differences observed in MCF-7^Var-H^ and MCF-7^Var-I^, mainly in relation to hormonal receptors expression, a proposal generated so far is that LDL overload achieved in MCF-7^VarI^ may have favored 27-hydroxy-cholesterol intracellular generation and ERα activation, allowing latency of this receptor.

Other significant changes were described in both variants, such as increased expression of the Nrf2 master regulator of redox homeostasis, but in a significant way in MCF-7^VarH^ (Figure 6A). We also observed increased nuclear localization in this cell variant (Figure 6B). It has been widely described that Nrf2 overexpression in different tumor types contributes to pro-oncogenic processes and chemoresistance [54] by promoting the transcription of antioxidant (NQO1), detoxifying (SULT1A1), and anti-apoptotic (BCL-2) enzymes with ARE element [55]. It has been described that Tamoxifen treatment can promote ROS generation, favoring Nrf2 activation and chemoresistance acquisition to therapy and other therapies in a concomitant way [56]. Interestingly, although both variants were generated by tamoxifen metabolites treatment, an increased expression of Nrf2 and augmented nuclear localization was observed predominately in MCF-7^VarH^. Inactive Nrf2 is mainly localized in cell membranes interacting with caveolin-1 (Cav-1) in lipid rafts. Lipid raft formation favored by high cholesterol concentrations, such as in MCF-7^Var-I^, could hinder Nrf2 nuclear translocation [57] and explain the observed behavior among variants (Figure 7).

Furthermore, differential expression of Nrf2-dependent genes was observed in both variants. Although the canonical Nrf2 pathway involves Nrf2-sMaf complex formation, other transcription factors could be influencing Nrf2 differential downstream genes expression among variants. It has been described that Nrf2 interacts physically and forms a complex with Activating Transcription Factor 4 (ATF4) promoting specifically HO-1 transcription, while Nrf2-ATF3 can act as a repressor of Glutathione S-transferase (GST) expression [58]. Even more, observed metabolic adaptation in variants could be related to Nrf2 action as well. It has been described that increased Nrf2 signaling is associated with suppression of lipogenesis related genes like FAS to avoid NADPH expenditure, necessary for detoxification reactions [59], which corresponds with what we observed in MCF-7^Var-H^.

Finally, Nrf2 could be implicated in chemoresistance adaptations in other ways that have not yet been described. Notably, under the subtle induction of ER stress by Tuni and FBS, which leads to an indirect Nrf2 activation, data suggest an inverse relationship between Tuni dose and hormonal receptor expression. This relationship has to be verified with more analysis.

Elucidating the role of LDL in tamoxifen chemoresistance development and the mechanism involved bring us closer to propose new strategies and/or pharmacological targets to prevent or counteract chemoresistance, avoiding complications in ER+ tumors treatment. Nrf2 inhibition is a promising therapeutic approach for Nrf2-dependent cancers and Nrf2-inhibitors are actively being developed in our group (personal communication).

## Conclusions

We evaluated the LDL involvement in chemoresistance acquisition to the two mainly active tamoxifen metabolites, endoxifen and 4-OHTam, by the development of two cellular variants derived from an ERα+ (MCF-7) cell line. We observed a completely different behavior when comparing variant MCF-7^VarH^ generated by tamoxifen metabolites treatment and variant MCF-7^VarI^ generated by co-incubation of tamoxifen metabolites and high LDL concentrations (75 µg/ml). Variants showed differences in the IC_50_ to metabolites, morphology, expression of hormone receptors, metabolic characteristics, and processes associated with metastasis. MCF-7^VarH^ showed similar features to those presented by triple-negative cells and increased antioxidant response, while MCF-7^VarI^ only showed subtle changes in its phenotype compared with MCF-7 cells. A better understanding of the metabolic changes displayed in these cellular variants allows us to propose new pharmacological targets, such as the master factor of the antioxidant response Nrf2, which can be modulated with current pharmacological treatments and potentially allow recurrence-free recovery in patients.

## Supplementary Material

Supplementary Figures S1-S9

## Data Availability

All data generated in this study is presented within the manuscript, supplementary material or could be request by e-mail.

## Abbreviations

4OH-Tam: 4-hydroxytamoxifen
ARE: antioxidant response element
ATF4: Activating Transcription Factor 4
BC: Breast cancer
Cav-1: caveolin-1
CHOL: cholesterol
IC_50_: Inhibitory concentration 50
E2: estradiol
Endoxifen: 4-hydroxy-N-desmethyl-tamoxifen
ER: endoplasmic reticulum
ERα: estrogen receptor α
FAK: Focal adhesion kinase
FAS: fatty acid synthase
FBS: Fetal Bovine Serum
GPR30: G protein-coupled receptor 30
GST: Glutathione S-transferase
HDL: high density lipoprotein
HER2: human epidermal growth factor receptor type 2
HO-1: heme oxygenase-1
LDL: low-density lipoprotein
LDLR: low-density lipoprotein receptor
MMP-9: metalloproteinase-9
NQO1: NAD(P)H quinone oxidoreductase 1
Nrf2: nuclear factor erythroid 2-related factor 2
PR: Progesteron Receptor
ROS: reactive oxygen species
SERM: selective estrogen receptor modulators
SULT1A1: Sulfotransferase 1A1
TNBC: triple negative breast cancer
Tum: Tunicamycin
UPR: unfolded protein response
