# Supplementary material for: Tamoxifen metabolites treatment promotes ERα+ transition to triple negative phenotype in vitro, effects of LDL in chemoresistance
Source: Biosci Rep. 2024 Aug 5;44(8):BSR20240444. doi: 10.1042/BSR20240444 (PMC11301570; doi:10.1042/BSR20240444)

Supplementary material

Sup. fig. 1. Characterization of VLDL/IDL, LDL, HDL, and plasma-free lipoprotein fractions; western-blot of apoB and ApoA-1 on the lipoprotein fractions

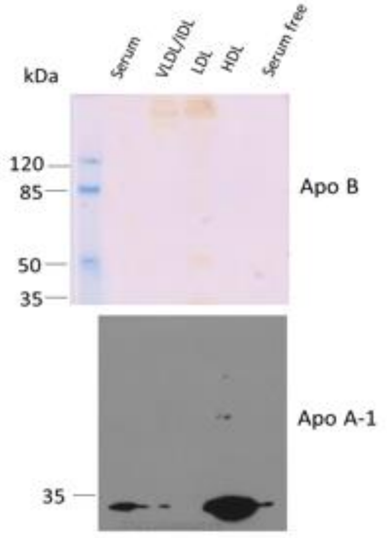

Sup. Fig. 2 Ki67 expression in MCF-7, MCF-7^{VarH}, and MCF-7^{VarI} cells under basal conditions. β-actin was used as a loading control.

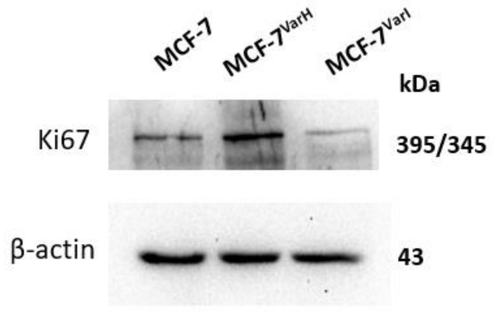

Sup. fig. 3. Densitometry analysis of SULT1A1 in MCF-7, MCF-7^{VarH}, and MCF-7^{VarI} cells under 4-OHTam or endoxifen metabolites (8 µM) under 24h. Results are reported as mean ± SD (n = 3) and expressed as % of control; * $p < 0.01$ respect to control. β-actin was used as a loading control.

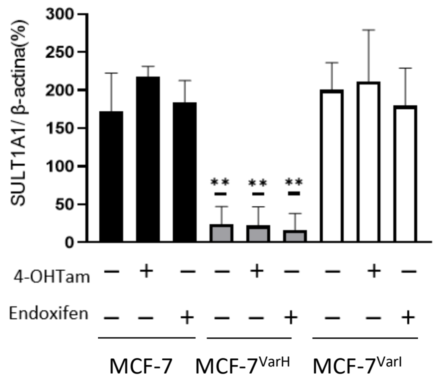

Sup. Fig. 4 Results of molecular docking assays of SULT1A1 versus tamoxifen, tamoxifen metabolites and estradiol. Protein Data Bank access code of SULT1A1: 4GRA.

**Metabolites**	**E-score (kcal/mol)**	**Residue Interactions**
Estradiol	-6.2995	Lys 48, Trp 53, Thr 227
Tamoxifen	-5.2633	Tyr 190, Lys 206, Phe 192
4-OHTam	-5.1393	Ala 101, Arg 72, Thr 99
Endoxifen	-5.7479	Arg 37, Gln35, Lys 97

Sup. Fig. 5 Nrf2 and hormonal receptor expression (ERα, HER2) in MCF-7 and MCF-7^{VarH} cells under 24 h treatment with increasing concentrations of Tunicamycin (Tuni). Western blot expression of Nrf2 in MCF-7^{VarH} cells (**A**) and MCF-7 cells (**C**) under a range of Tuni concentrations (0.25-4 µM). At the same conditions, ERα and HER2 expression in MCF-7^{VarH} (**B**) and MCF-7 (**D**) cells. β-actin was used as a loading control.

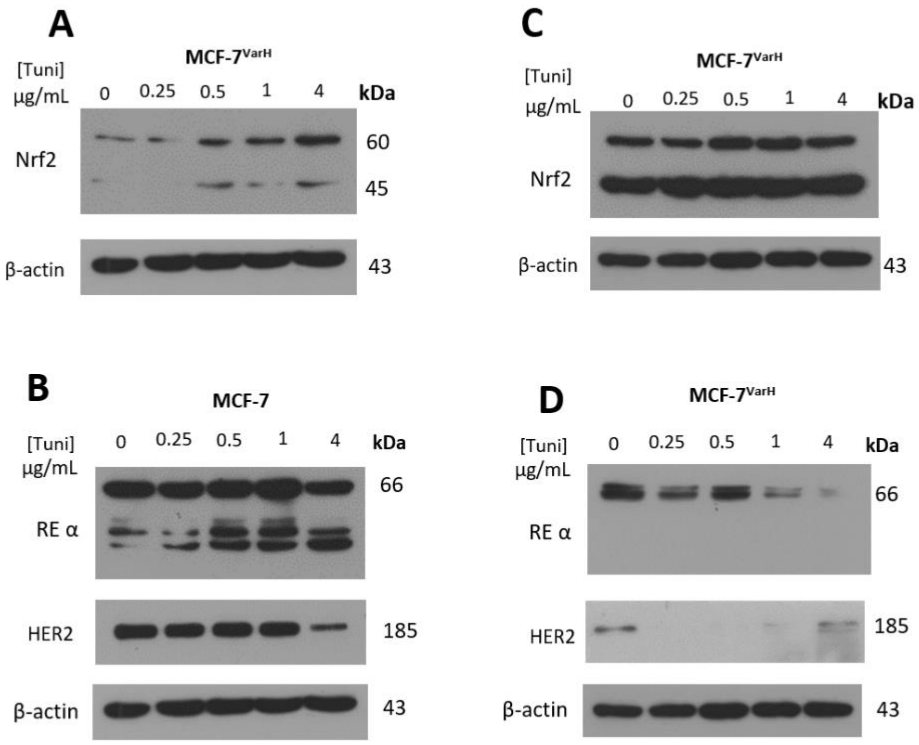

Sup. Fig. 6. Complete unedited blots from figure 3.

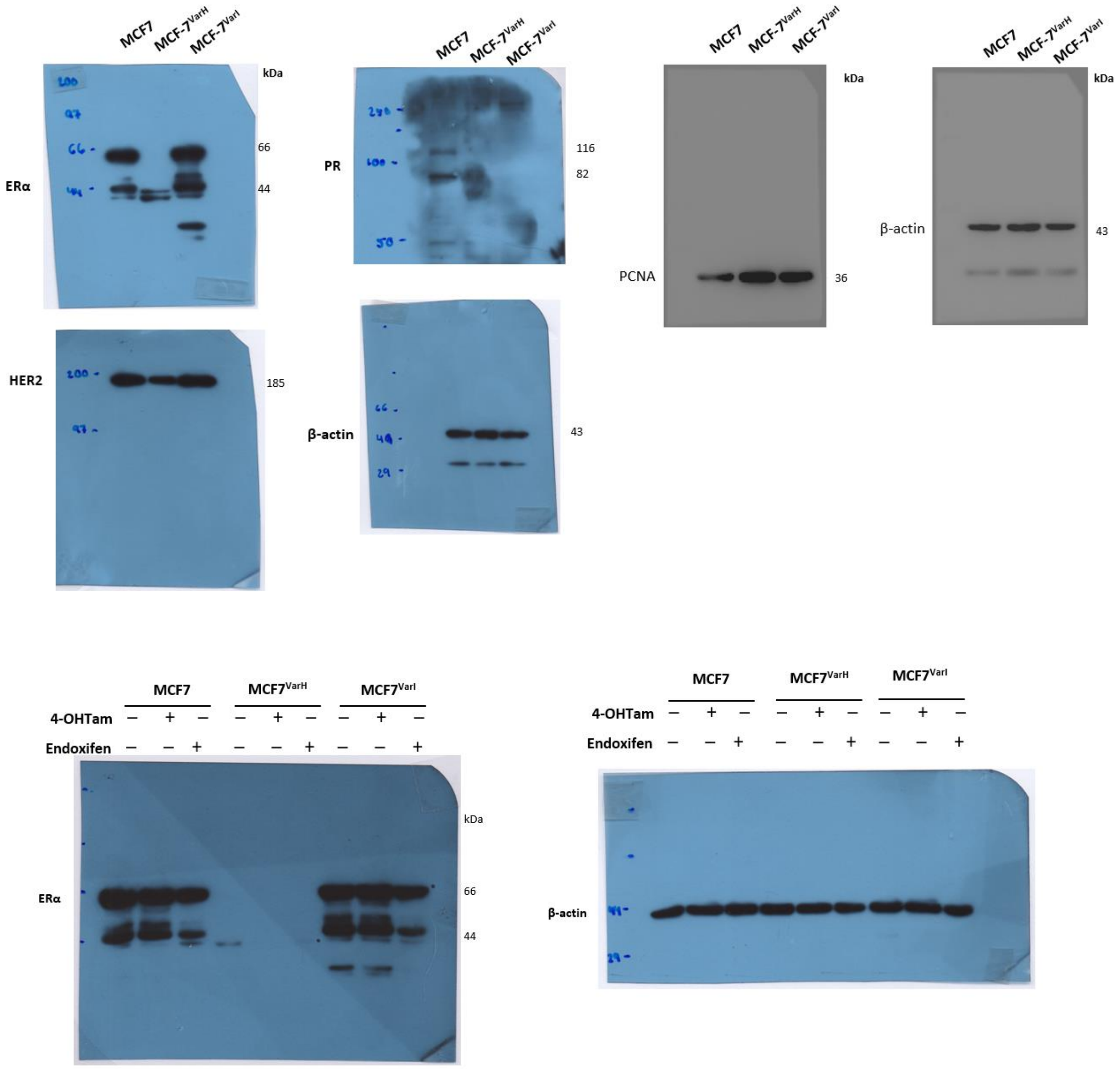

Sup. Fig. 7. Complete unedited blots from figure 4.

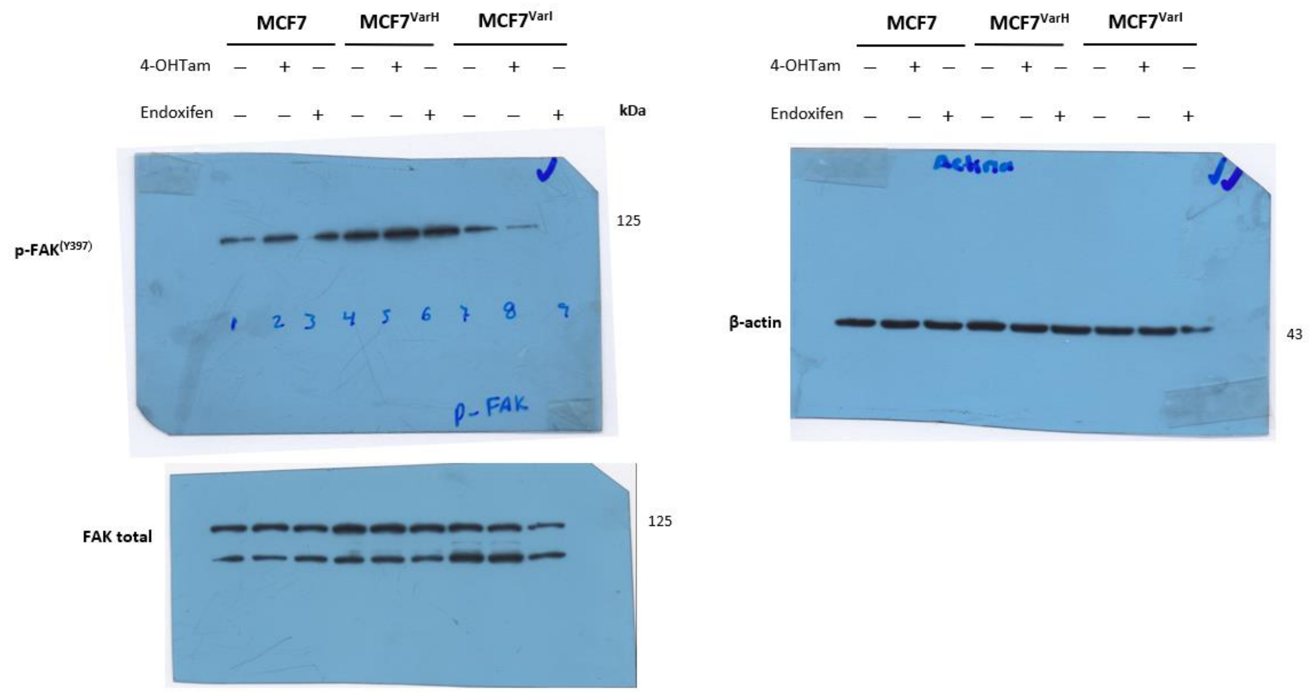

Sup. Fig. 8. Complete unedited blots from figure 5.

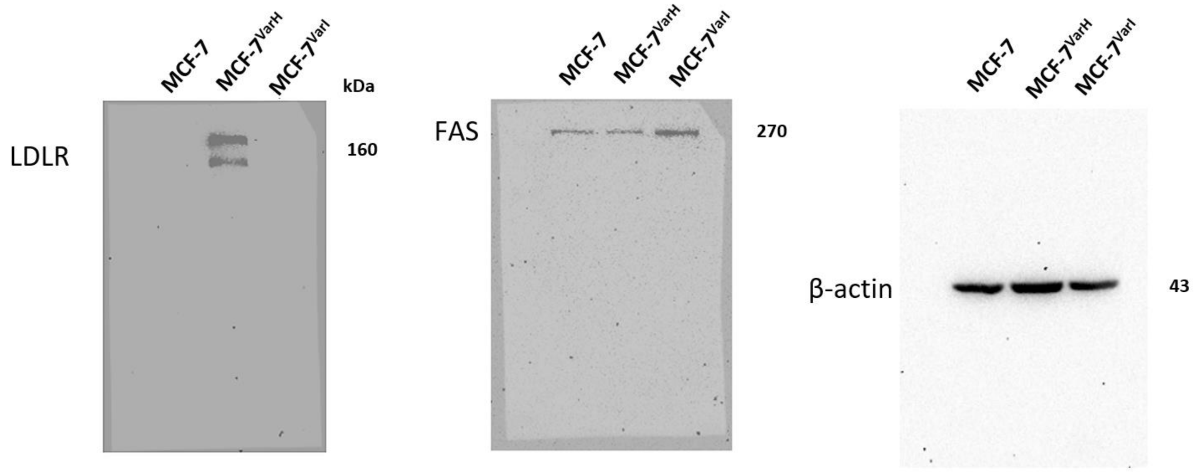

Sup. Fig. 9. Complete unedited blots from figure 6.

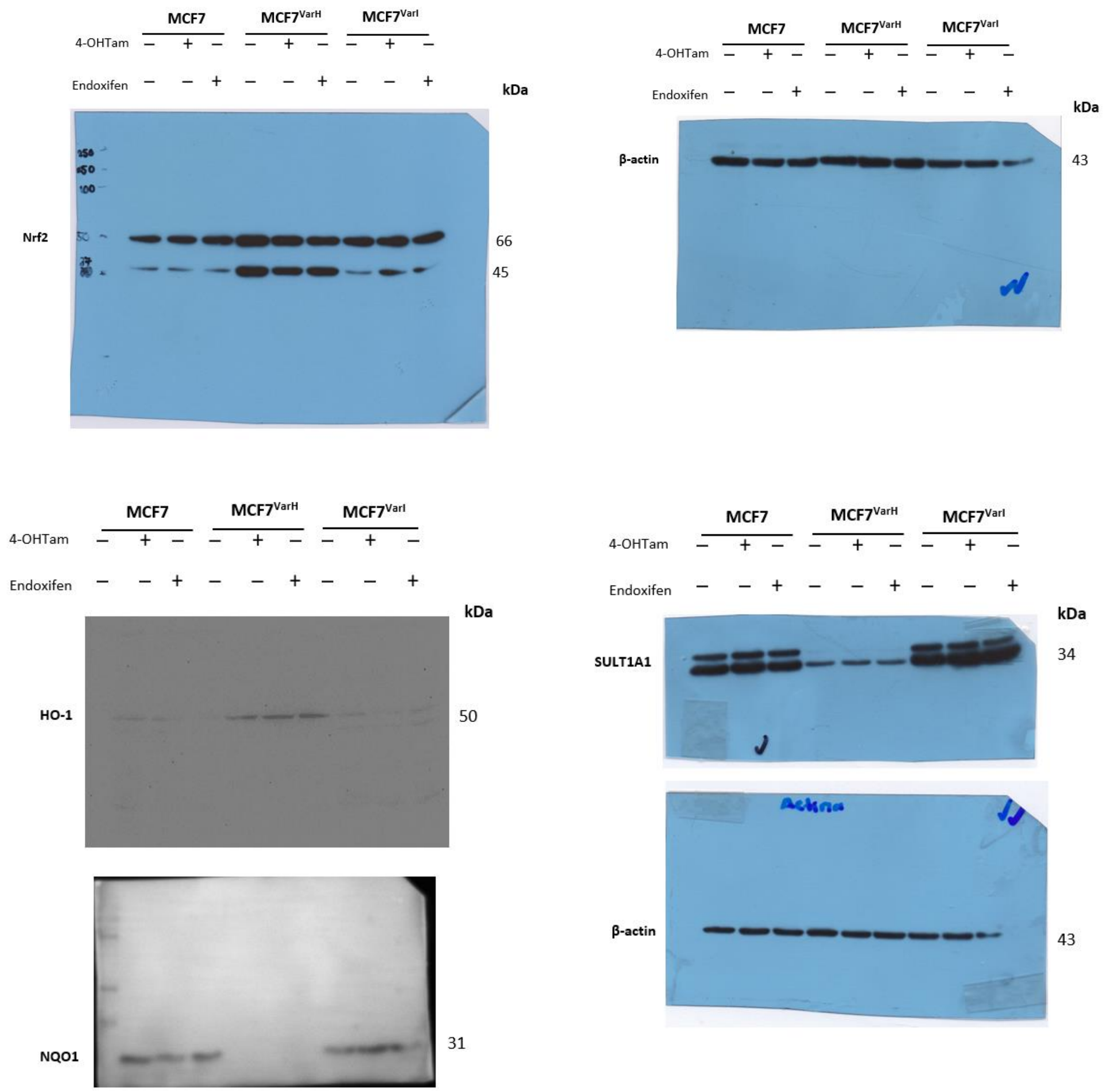

Supplement: Supplementary Figures S1-S9 [file BSR-2024-0444_supp.pdf]
